# THE EFFECTIVENESS OF A PEER-BASED INTERVENTION ON SOCIALLY ISOLATED OLDER CHINESE IMMIGRANTS IN CANADA

**DOI:** 10.1093/geroni/igz038.1347

**Published:** 2019-11-08

**Authors:** Daniel, W L Lai, Jia Li, Liza Chan, Celia Li, Alison X Ou

**Affiliations:** 1 The Hong Kong Polytechnic University, Hong Kong, Hong Kong; 2 Calgary Chinese Elderly Citizens’ Association, Calgary, Canada; 3 Calgary Chinese Elderly Citizens’ Association, Calgary, Alberta, Canada

## Abstract

Objectives: This study aimed to examine the effectiveness of a peer-based intervention in reducing older Chinese immigrants’ loneliness and social isolation to improve their psychosocial well-being. Method: A randomized controlled trial design was adopted. A sample of 60 community-dwelling older Chinese immigrants aged 65 and above were randomly assigned to the intervention group (n=30) and the control group (n=30). Intervention group participants received an eight-week peer support intervention. 25 volunteers aged 48 to 76 were recruited and trained to provide one-to-one peer support services through home visits and telephone. The services included multiple activities such as providing emotional support, assisting in problem-solving, and community resources sharing. Different types of activities were consecutively executed throughout the eight weeks in accordance with the service protocol. Standardized assessments including loneliness, social support, social participation, and other psychosocial outcomes such as life satisfaction, happiness, depression, and resilience at baseline and after intervention were measured. Results: After the intervention, as compared to control group participants, intervention group participants reported a significant decrease in loneliness, fewer barriers in social participation, and a significant increase in resilience. They also reported fewer depressive symptoms, increased life satisfaction, and happiness, but no such improvements were observed in the control group. Discussion: The study findings illustrate the need to further examine the use of peer-based interventions for both program effectiveness and delivery efficiency. In the era of population aging, baby boomers can be trained to take up more volunteer roles to serve older adults in distress via peer-based intervention approaches.

